# High purity and viability cell separation of a bacterivorous jakobid flagellate based on a steep velocity gradient induced soft inertial force†

**DOI:** 10.1039/c8ra05328f

**Published:** 2018-10-16

**Authors:** Pan Deng, Cheng-Jie Fu, Zhigang Wu

**Affiliations:** State Key Laboratory of Digital Manufacturing Equipment and Technology, Huazhong University of Science and Technology Wuhan China zgwu@hust.edu.cn zhigang.wu@angstrom.uu.se; Department of Organismal Biology, Uppsala University Uppsala Sweden chengjie.fu@gmail.com; Department of Engineering Sciences, The Ångström Laboratory, Uppsala University Uppsala Sweden

## Abstract

Cell separation is one of the key limiting factors for precise analysis of non-axenic microbial lab cultures or environmental samples, and it remains a challenge to isolate target cells with high purity and viability *via* high-throughput cell sorting. During the past decade, hydrodynamic microfluidic platforms have attracted great attention in cell preparation for their high efficiency, robust performance and low cost. Here, we employ the use of a low-velocity sheath flow with high viscosity near the wall and a high-velocity sheath flow with low viscosity on the other side of the sample flow in a soft inertial separation chip. This not only prevents hard interactions between cells and chip walls but, in comparison to previous inertial separation methods, generates a significant increase in deflection of large cells while keeping the small ones in the original flow. We first conducted experiments on a mixture of small and large fluorescent particles (1.0 and 9.9 μm, respectively) and removed over 99% of the small particles. The separation efficiency was then tested on a culture of a bacterivorous jakobid flagellate, *Seculamonas ecuadoriensis* fed on the live bacterium, *Klebsiella* sp. Using our microfluidic chip, over 94% of live bacteria were removed while maintaining high jakobid cell viability. For comparison, we also conducted size-based cell sorting of the same culture using flow cytometry, which is widely used as a rapid and automated separation tool. Compared with the latter, our chip showed more than 40% higher separation efficiency. Thus, our device provides high purity and viability for cell separation of a sensitive cell sample (jakobid cells). Potentially, the method can be further used for applications in diagnostics, biological analyses and environmental assessment of mixed microbial samples.

## Abstract 2

Aimed at separating living cells with high purity and viability from non-axenic microbial lab cultures or environmental samples, we developed a novel microfluidic separation technique with simple operation, high efficiency and robust performance.

## Graphical abstract

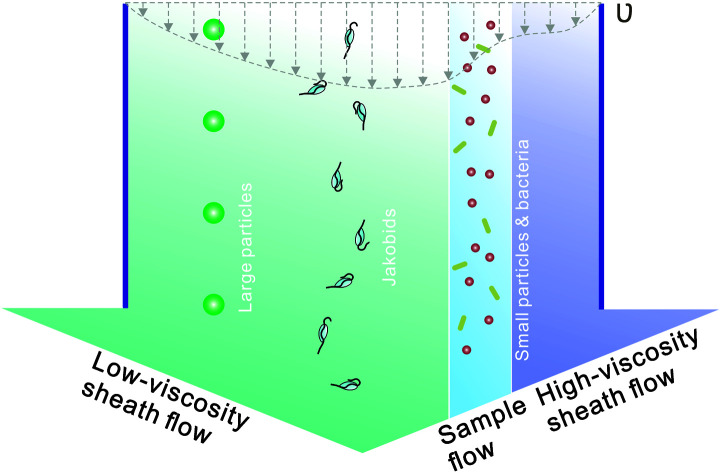

## 1. Introduction

Cell separation is a pivotal step in sample preparation for a wide range of biomedical and environmental applications. For instance, in many laboratory experiments, cell disruption during purification procedures leads to the loss of valuable and often very limited samples. For example, in commercial exploitation of algae production, detecting adventitious organisms at the initial stage could enable operation engineers to take timely measures to avoid large-scale contamination and thus reduce economic loss.^1^ Such tasks require both high purity and high viability of a purified cell population to allow precise diagnostics and analyses of the target cells.^2^

Achieving high cell purity and viability *via* high-throughput separation techniques is particularly demanding and challenging.^3^ Currently-used conventional methods for environmental cell collection are either based on natural sedimentation, dilution, or capillarity, which are time-consuming, or based on pH and temperature, which compromise sample viability due to their sensitivity to environmental change.^4^ Since microbial cell dimensions are well suited for the channel sizes found in microfluidic devices, microfluidic chips have attracted great attention and numerous separation techniques have been developed employing them.^5–8^ These techniques are used, for example, to isolate circulating tumor cells for metastatic diseases detection and characterization,^9,10^ to separate blood cells to assess human health,^11–13^ or to sort harmful bacteria relating to food-borne diseases^14,15^ or pathogenic microorganisms.^16^ However, little work has been done exploring the potential of microfluidic separation for the preparation of environmental samples.^17^ This is an important field in many respects, particularly as most microbial diversity, both prokaryotic and eukaryotic, is uncultured but nonetheless ecologically, medically and evolutionarily important.

Jakobids are free-living heterotrophic flagellates, probably common in many aquatic systems. These eukaryotic cells feed primarily on bacteria captured by an unusual and possibly ancient suspension feeding system. Jakobids are of particular evolutionary interest due to their strikingly bacterial-like mitochondrial DNAs (mtDNA). These are thought by many to hold the key to understanding the origin of this critical eukaryote-defining organelle, sometimes referred to as the powerhouse of the eukaryotic cell.^18^ However, a recent study indicated that jakobid mtDNA was actively acquiring DNA directly from bacteria, a process known as horizontal gene transfer (HGT).^19^ This suggests a surprising alternate explanation for the bacteria-like jakobid mtDNA, and a previously unsuspected molecular promiscuity for this important eukaryotic organelle. Further understanding of the functional significance and probable physical mechanism of mitochondrial HGT requires working with purified cells in technique such as mitochondrial proteomics. However, bacterial contamination during jakobid mitochondria preparation would interfere with the subsequent biochemical analysis. Thus, obtaining a large amount of highly pure and viable jakobid cells is critical to these lines of research.

Size difference is a quite common and straightforward property for microfluidic separation, and a variety of techniques have been developed, *e.g.* filtration,^11,20^ inertial fractionation,^21,22^ pinched flow fractionation (PFF),^23^ dielectrophoresis system^24^ and optical tweezers.^25^ Continuous inertial fractionation with sheath flows is particularly attractive for its simple structure, free-external-energy field, potential high throughput and soft interaction between particles and flows.^26,27^ Isolating living cells with dynamic morphological shapes is particularly challenging since the deformability of soft cells could weaken the effect of inertial force.^28–30^ Such problems could be worse when the target cells are asymmetric like the dorsoventrally organized jakobids.

**Fig. 1:**
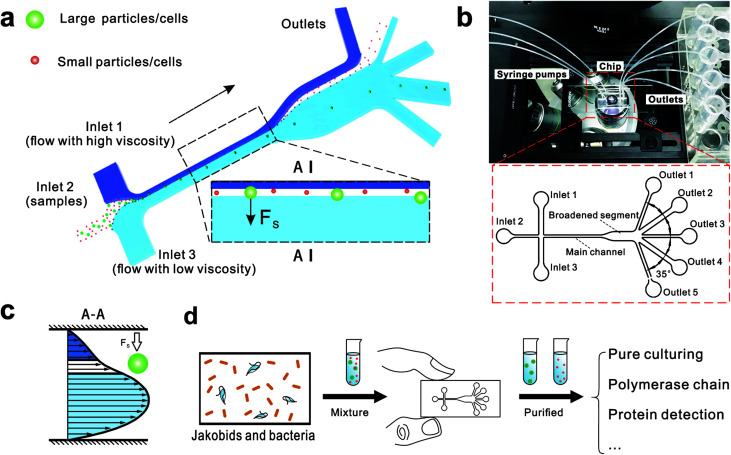
The design and potential usage of the microfluidic chip for environmental sample separation. (a) Schematic of microfluidic chip for particle separation and collection illustrating the principle of particle/cell sorting. (b) Photograph of the microfluidic chip fixed under a microscope and integrated with three syringe pumps and five tubes for outflow collection. (c) The fluid velocity profile at A–A place. (d) Potential use of microfluidic chip for high-throughput cell sorting of environmental samples.

Here, we describe a novel microfluidic separation technique aimed at separating living eukaryotic and bacterial cells with higher purity and higher viability as compared to conventional methods. The method was tested on mixtures of fluorescent synthetic particles and then on a mixed culture of living jakobid cells and their bacterial food, *Klebsiella* sp. A steep velocity gradient was generated using large differences of two sheath flows in both velocity and viscosity (Fig. 1a and c). This gives rise to a lift force that in turn leads to greatly enhanced lateral migration of larger particles.^31^ This method was first tested on a mixture of fluorescent particles with diameters of 9.9 and 1.0 μm, whose lateral distances in the broadened segment could serve as a reference for subsequent live cell separation. The method was then used to acquire jakobid cells with high purity and high viability from the bacteria *Klebsiella* sp. The separation results showed that our method outperformed a widely used conventional biotic separation technique in terms of cell purity under the prerequisite of high cell viability. Thus, our separation chip using this microfluidic method described here has potential utility in some important applications such as environmental sample purification. For example, it should be able to separate combinations of symmetric and asymmetric cells like *Chlorella* and *Lactobacilli*, as well as fragile and sensitive cells (Fig. 1d).

## 2. Theoretical background and characterization

Fig. 1 shows the schematic of microfluidic chip and working principles of separation. The rate of fluid flow through the microchannel is analyzed by Reynolds number (Re) and particle Reynolds number (Re_p_), which are defined as the ratio of inertial force to viscous force,1
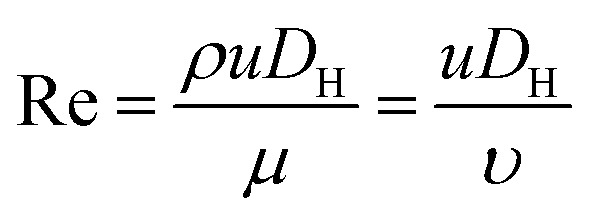
2
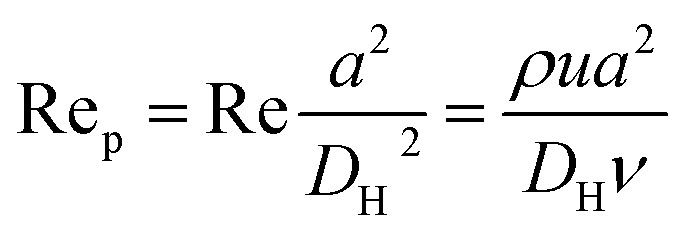
where *ρ* is the density of the fluid, *u* is the average velocity of the fluid, *μ* is the dynamic viscosity of the fluid, *υ* is the kinematic viscosity of the fluid, and *D*_H_ is the hydraulic diameter of the microchannel, and *a* is the diameter of the particle in the sample. When the particle Re is much less than 1, there is an assumption that the particle is driven by viscous drag and does not disturb the underlying flow field. Peclet number (Pe) is used to describe the motion of particles in the fluid, and is defined as the ratio of the rates of diffusion *versus* convection of particles:^32^3
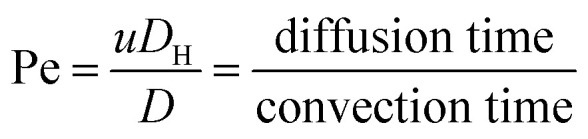
where *D* = *kT*/3π*ηa*, with *k* and *T* being the Boltzmann constant and the absolute temperature, respectively. According to this formula, smaller particles will lead to low Pe and thus be more likely to be diffusive along the microchannel. Lower flow rate can also reduce the diffusive effects. For a particle in the accelerating flow, the Stokes number (St) is a measure of the particle fidelity to the flow. St is defined by the particle relaxing time (*τ*_r_) and the characteristic time of the flow (*τ*_f_) as follows:^33^4
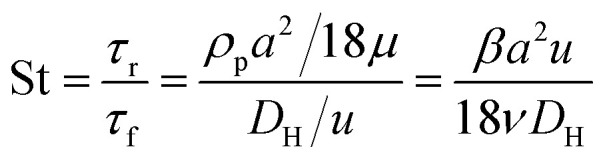
where *β* = *ρ*_p_/*ρ* and *ρ*_p_ is the density of the particle. Particles show better tracing accuracy and faster response to the flow rate when the fluid is in acceleration, *i.e.*, the Stokes number approaches zero.

The behavior of non-neutrally buoyant particles is dominated by a lift force with Re_p_ = 1 and low Re in Poiseuille flow. The dominant lift force on particles is the Saffman's force^34^ (*F*_s_), which is caused by the interaction of the Stokeslet velocity field:

5 *F*_s_ = *KUa*^2^(*γ*/*ν*)^1/2^

where *K* is a constant (*K* ∼ 6.46) obtained by numerical integration, *U* is the relative velocity of particle and fluid measured along the streamline through the center of particle, and *γ* is the fluid velocity gradient. The effect of this Saffman's force determines the direction of movement and preferred location of particles. This leads to the possibility of separation when particles have different sizes (Fig. 1a).6
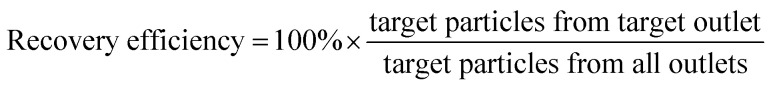
7
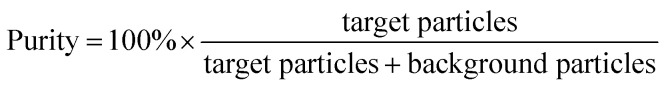


## 3. Experimental

### 3.1 Jakobid cell culture and treatment

Cells of the jakobid, *Seculamonas ecuadoriensis* (ATCC no. 50688), were grown in WCL culture medium^35^ in 2 liter Fernbach flasks. Cultures (500 ml) were fed with pre-cultured live bacteria (*Klebsiella* sp.) and shaken on a rotary shaker (80 cycles per min) at room temperature. Cells were harvested in early stationary growth phase in 50 ml corning tubes by a 10 min centrifugation at 800×*g*, when jakobid cells reached a density of approximately 4.5 × 10^5^/ml. For antimicrobial treatment of bacteria, lysozyme (Sigma-Aldrich) (0.4 mg ml^−1^) and lactoferrin (Sigma-Aldrich) (0.1 mg ml^−1^) were added to harvested cell samples 1 h before cell sorting. Methylene blue dye (Sinopharm Chemical reagent) was diluted 1 : 100 in double-distilled water for cell viability test.

### 3.2 Microfluidic chip design and fabrication

The microfluidic chip was fabricated using a standard soft lithographic technique. First, the silicon wafer, purchased from Tebo (Harbin, China) was prepared in three RCA clean steps (RCA I, RCA II and RCA IV)^36^ to wash off organic residues and metallic contaminants. Then the silicon wafer was coated with a negative photoresist SU-8 3025 (Micro Chemie, MA, USA) and spun at 3500 rpm to obtain a uniform thickness. After soft baking at 95 °C for ten minutes, the wafer was exposed to a UV light under a plastic photo mask and then post baked at 95 °C for four minutes. The same developer (Micro Chemie, MA, USA) was used to get the desired channel network patterned onto the wafer. In order to obtain stable and hard photoresist, the resist was hard baked for 30 min at 150 °C. From this master, replicas were molded using a mixture of PDMS prepolymer and a curing agent (9 : 1 by weight) (polydimethylsiloxane, Wacker Chemie, München, Germany), which was then poured onto the patterned wafer and cured for 15 min at 75 °C. The PDMS was then peeled off from the master and bonded to a glass slide (Citoglas, China) after surface treatment using a corona discharger (BD-50E, Electro-Technic Products Inc., IL, USA).

The chip design is shown in Fig. 1, including three inlet channels, a major channel, a broadened segment and five outlet channels. The entire microchannel is 24 μm high. The Inlet 1 and Inlet 3 have a width of 120 μm, and Inlet 2 has a width of 50 μm. The width and length of the main channel are 50 μm and 5700 μm, respectively. There are three identical narrow outlet channels (Outlet 1, Outlet 3, Outlet 5) for waste and two identical wide outlet channels (Outlet 2, Outlet 4) for target particles whose widths are 300 μm and 500 μm, respectively. The angle between two neighboring outlet channels is 35°.

### 3.3 Device and fluid preparation

To prevent particles from adhering to channel walls, the microfluidic chip was filled with 1.5% hydroxypropyl cellulose (HPC) dissolved in 2-(*N*-morpholino)ethanesulfonic acid monohydrate (MES)/Tris(hydroxymethyl) aminomethane (TRIS) buffer (80 mM/40 mM) at 4 °C in a humid environment overnight. HPC, MES, and TRIS were supplied by Alfa Aesar, Karsruhe, Germany. After surface treatment using the corona discharger, all disposable syringes (Gemtier, Shanghai, China) were filled with 10% (w/w) Pluronic F-127 (SigmaAldrich, St. Louis, USA) dissolved in deionized (DI) water (MilliQ, Millipore Corp., USA) and stored at the same condition as the microfluidic chips.^27^

For synthetic particle preparation, fluorescent microbeads (Alfa Asear, Karlsruhe, Germany) with diameters of 1 μm (red fluorescent) and 9.9 μm (green fluorescent) were mixed at 1 : 5 by volume. 0.1% (w/w) bovine serum albumin (BSA) (REGAL, Shanghai, China), phosphate buffered saline (PBS, BasalMedia, Shanghai, China), and 1% (w/w) Pluronic F-127 were added to the mixture of microbeads to keep the microbeads from agglomeration.^27^ The high-viscosity flow was obtained by blending 23.5% glycerol (Sinopharm, China), 1% (w/v) Pluronic F-127, and 10% (v/v) Tween 20 (Sinopharm, China) solution with PBS solution, while the low-viscosity flow consisted of pure PBS solution.^31^ All fluids were driven by a syringe pump (PHD2000, Harvard Apparatus, Boston, USA). The movement of particles was monitored in dark using an inverted fluorescent microscope (Nikon Ti-u, Tokyo, Japan) with a color SLR camera (Canon EOS 70D, Tokyo, Japan), while high-speed movement was captured by a high-speed camera (Phantom V1212, Vision Research Inc, USA). Counting of particles or cells used a hemocytometer (Qiujing, Shanghai, China) with an inverted fluorescent microscope (Dmi8, Leica, Germany), and images were obtained using a monochrome digital camera (DFC365 FX, Leica, Germany).

### 3.4 Flow cytometer and cell sorting

Flow cytometer cell sorting was performed at the Microbial Single Cell Genomics Facility (SiCell), SciLifeLab. Prior to cell sorting, calibration was performed using 10 μm fluorescent beads. The harvested cell suspensions were filtered through BD Falcon Cell-Strainer Caps (# 352235). Samples were sorted at either room temperature or 5 °C on a Beckman Coulter Astrios^EQ^ cell sorter using a 488 nm laser for excitation, 100 μm nozzle, sheath pressure of 60 psi (normal) or 25 psi (low), and PBS solution as sheath fluid (0.2 μm filtered before use). Sorting of cell subpopulations of different sizes was based on a gating strategy relying on forward scatter (FSC) *versus* side scatter (SSC) parameters. Cell fractions were sorted into 0.5 ml Eppendorf tubes containing 20 μl of WCL culture medium using the single cell sorting mode.

## 4. Results and discussion

### 4.1 Particle separation performance

**Fig. 2:**
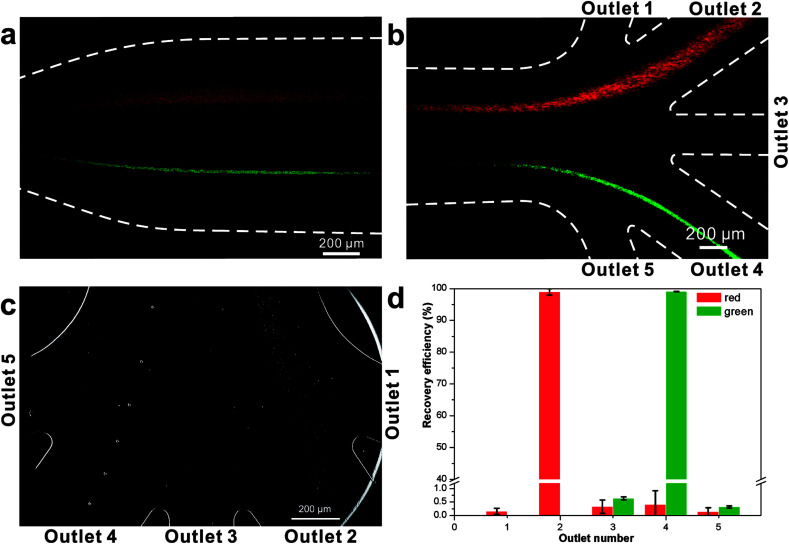
Photograph of separated particles at the broadened segment near the entrance (a) and near the outlets (b and c) of the microfluidic separation chip. The smaller particles (diameter 1.05 μm, red fluorescent) were collected at Outlet 2, while larger particles (diameter 9.9 μm, green fluorescent) were collected at Outlet 4 (Movie S2†). The separation was run with a sample flow rate of 2 μl min^−1^ (d).

**Fig. 3:**
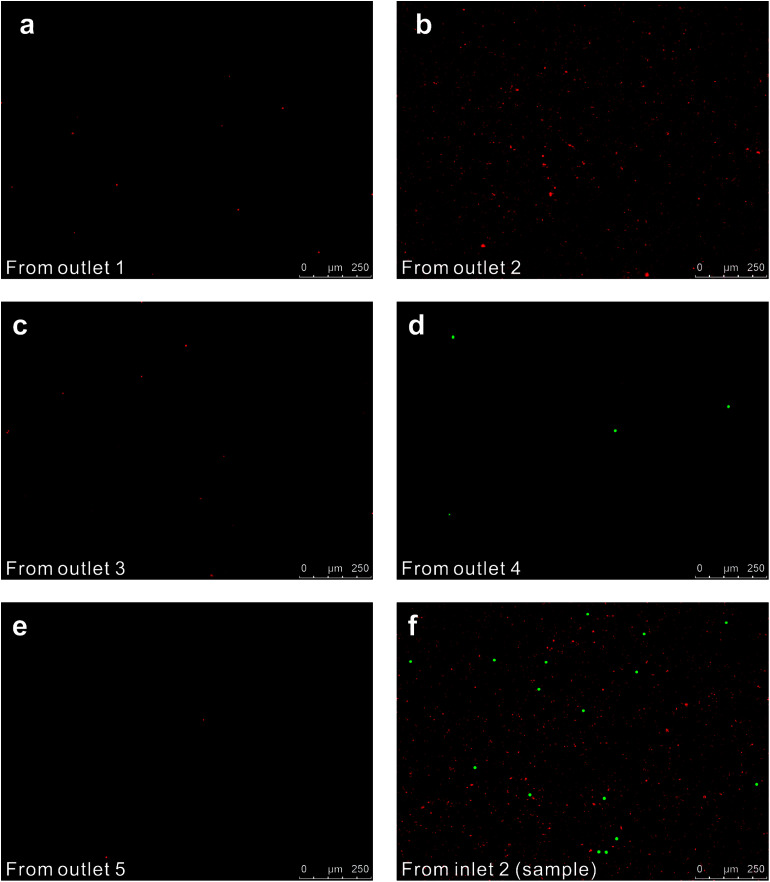
Fluorescent particles as seen in a hemocytometer under the microscope using a pseudo color method: (a), (c), and (e) show waste collected from Outlet 1, Outlet 3 and Outlet 5, respectively; (b) and (d) show red and green particles collected after separation from Outlet 2 and Outlet 4, respectively; (f) original sample shown at 10 times dilution.

We initially evaluated the particle separation efficiency of our custom-designed microfluidic chip using two different-colored fluorescent particles which differed in diameter of 1 μm (red) and 9.9 μm (green) (Fig. 1b). Three inlets (Fig. 1a) were connected with a sheath flow with high viscosity, a sheath flow with low viscosity and a sample flow, corresponding to flow rates of 5 μl min^−1^, 30 μl min^−1^ and 2 μl min^−1^ (Re < 1), respectively. The Outlets 1, 3 and 4 were used to collect waste solution, while Outlets 2 and 5 collected the target particles. When particles were focused along the main channel (Fig. 1c), steep velocity gradient tends to accelerate (lift) the migration of particles towards the flow with lower viscosity.^34,37^ In order to push particles with different sizes widely apart, we tried to magnify the difference in *F*_s_ by increasing *γ* (eqn (5)). The sharp difference between two sheath flows in *u* and in *υ* generated a steep velocity gradient, and it reinforced *F*_s_ directed towards the higher relative velocity side^37^ (Fig. 1c). *F*_s_ is positively correlated with particle size, so the small particles (red) are driven closer to the wall side while the large particles (green) are deflected and cross the centerline of the channel (Fig. 2). The strongly size-dependent St (eqn (4)) could also explain why small particles show a strong preference to follow the original carrier fluid. As demonstrated in Fig. 2a, the particles' difference in positions is amplified in the broadened segment of the chip. Moreover, small particles exhibited dispersion (Movie S1†) due to small Re_p_ and Pe. High-speed camera shows more precisely the large dispersion distance between the large and small particles (Fig. 2c). The numbers of particles collected from each of the five outlets were counted using a hemocytometer under an inverted fluorescent microscope. Particle recovery efficiency was defined taking into account the deposition effect of particles in the syringes (eqn (6)). The resulting recovery efficiency was nearly 99% for small particles and above 99% for large particle (CV < 1.1%) (Fig. 2d). The purity (eqn (7)) of the output particles was also analyzed under a microscope using a sample collected after 20 minutes. This showed no green particles in Outlet 2 (Fig. 3), indicating nearly 100% purity of small particles. Due to the small flow rate and smaller size, diffusion dominated (Pe) and small particles were more likely to experience a lateral dispersion vertical to the streamline. As a result, a few small particles were found in the waste outlets and in Outlet 4 (Fig. 3a and c–e), and altogether these comprised about 1% of the total of small particles. Before the separation, the ratio of small particles to large particles was about 2000 : 1 (Fig. 3f), while more than 99% of small particles were separated from large particles counted in Outlet 4. Thus, compared to the sample solution, the large particles were diluted about 47 times while the small particles were diluted less than 4.6 times. Since the distribution of particles was consistent, higher density of collected particles could be achieved using a smaller width of target outlet channels or a higher flow rate of the sample solution. As indicated in our previous^31^ and current study, high separation efficiency of particle can be achieved when a sheath flow with a viscosity roughly twice that of another sheath flow. Higher throughput for cell separation should be achievable by increasing flow rate ratio or viscosity ratio between two sheath flows.^31^ However, high shear gradient may cause damage for fragile or sensitive cells during the focusing, where further biological verifications are needed.

### 4.2 Cell separation tests of a jakobid flagellate and its bacterial food

Jakobid flagellates are fairly slow swimming suspension predators with a relatively low growth rate and they likely require relatively high concentrations of their bacterial food.^38^ We used fresh cultured, live bacteria (*Klebsiella* sp.) as a single-bacterium food source in culture, although the culture probably also contained a few other microbes carried-over from the original jakobid environmental sample. Unlike the test particles (above), jakobid cells are roughly ellipsoidal (Fig. S1a†) with an average long axis of 14.7 μm and minor axis of 6.4 μm (in static free environment). Jakobids also show a finite deformation capacity when temperature or extracellular pressure changes and especially under shear stress conditions. Compared to normal cells (in static free environment), jakobid cells pushed through a microchannel (in dynamic constrained environment) were smaller and thinner due to the flow pressure and shear stress (Fig. S2†). These differences between live cells and synthetic particles probably impairs the separation efficiency for cells relative to the particles.

**Fig. 4:**
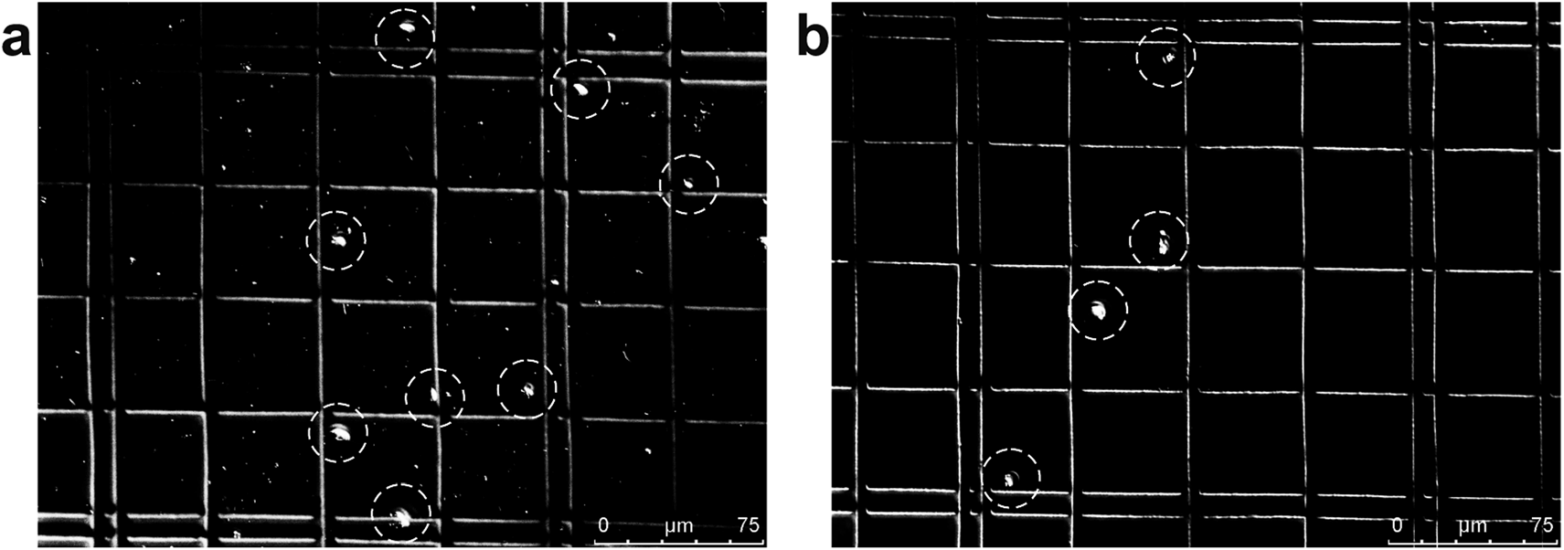
Images of jakobid cells in a hemocytometer under the microscope: (a) the sample before separation – the larger brighter dots are jakobid cells and the smaller ones are bacteria; (b) purity of bacterial cells collected from Outlet 3.

Fig. 4 shows an example of separation performance for jakobids and bacteria. Jakobid cells were collected and then analyzed by a hemocytometer. The hemocytometer used for cell counting had a standard depth of 100 μm, and the side length of the smallest counting chamber (square) was 40 μm. The different physiological states of the jakobid cells and bacteria made the jakobid cells appear larger and brighter (Fig. 4). Unlike the green fluorescent particles (9.9 μm), which were recovered from Outlet 5 (see above), most jakobid cells were recovered from the Outlets 2, 3 and 4, with a recovery efficiency of 67.3%, 19.2%, and 13.4%, respectively. At the same time, the recovery efficiency of the bacteria in Outlets 3 and 4 was 5.5%, and 3.6%, respectively. The jakobid cells did not deflect as far as the green fluorescent particles and the bacteria did not stay as well in the sample flow as the red fluorescent particles under the same flow conditions. Therefore, it should be considered whether the priority is to be cell purity or recovery efficiency to optimize the results, since higher recovery efficiency might be at the cost of increasing the risk of cross contamination.^27^ In order to obtain jakobid cells with both high purity and good recovery, Outlet 3 was chosen as the target outlet, as the jakobid cells collected from it were only diluted 2.2 times compared with the original density in the sample flow.

**Fig. 5:**
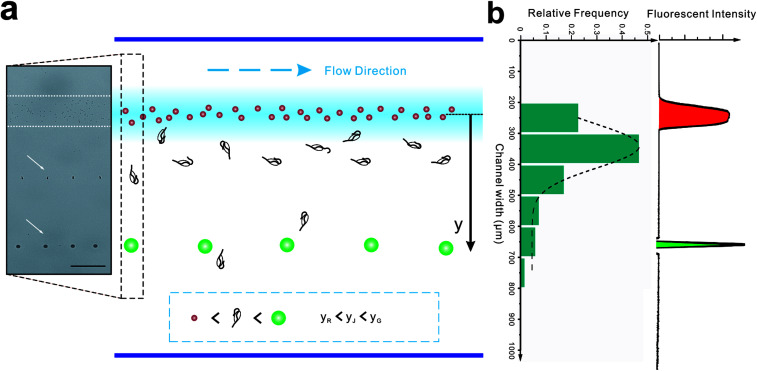
Distribution of synthetic particles and live jakobid cells in the broadened segment. (a) The schematics of positions of particles and cells with a high-speed image showing the trajectories of a green particle and a jakobid cell (the midline of the carrier flow in broadened channel was selected as the origin). (b) Experimental histogram of jakobid cells (*N* = 70) and grey-scale profile of particle trajectories.

The lower purity and recovery efficiency obtained from live cells *versus* synthetic particles could be due to the different relative sizes of bacteria and jakobid cells compared with those of the small and large synthetic particles. Also, the size of jakobid cells may vary among due to individual differences in morphology, growth stage, deformation, and/or rotation. The small size of the rod-shaped bacteria (0.3–1.5 μm wide by 0.5–5 μm long) could also have caused them to be more likely to rotate than jakobid cells. If so, this would cause the bacteria to present different postures in the main channel, resulting in varied inertial forces. However, as long as the bacteria with the largest inertial force or the smallest with the highest diffusivity could not escape from the sample flow and flowed into the Outlet 2, highly pure jakobid cells could still be acquired from other outlets. For jakobid cells, both deformation due to the shear stress^39,40^ and size inhomogeneity due to different growth stages could cause its large and uneven dispersion (Fig. 5). Also, the minimum lateral displacement is generally determined by the minimum dimension of this kind of non-uniform shaped cells when distributed close the side of bacteria. Hence, an outlet wider than the cells' lateral dispersion could provide a higher recovery efficiency of jakobid cells. Moreover, as aforementioned, by increasing flow rate ratio of two sheath flows or using higher viscosity sheath flow to decrease focusing width to the dimension close to the size of larger cells, they could be focused to a line and greater pressure difference between their two opposite sides could lead them migrate faster towards channel centerline than smaller ones.^31^ However, flow rates and viscosity should be controlled carefully to avoid harming fragile cells. In sum, the size of bacteria plays an important role in collecting pure jakobid cell, and the outlet configurations and changes made in flow rates or viscosity could substantially be used to optimize separation efficiency.

Introducing a co-flow system of sheath flows with different viscosities^31^ into “hard” inertial focusing and particle separation, Lee *et al.*^22^ demonstrated a particle separation with adjustable focusing positions. However, the particles need to pass through a long channel before finishing this long-term focusing process. Meanwhile, particle deflection in our work is a short-term momentum change which could be completed within a short channel. According to the theory of Saffman,^41^ the particle lateral velocity can be determined by the relationship of the particle diameter, shear rate and relative velocity.^42^ Assuming that the particle goes through the channel at a constant speed and with an identical relative velocity, the particle position in the broadened segment can be expressed as follow (Fig. 5b):

8 *y* =*β*_1_*β*_2_*a*^2^

where *y* is the particle position from the carrier flow, *β*_1_ is a constant coefficient, and *β*_2_ is a wall restriction determined coefficient. Theoretically, larger particles should deflect further away from the carrier flow and go closer to the wall, so the wall restriction is greater and *β*_2_ should be smaller.

To test this, the colored fluorescent particles (red and green) and jakobid cells were mixed in the sample flow, for which their mean positions in the carrier flow in the broadened segment are 10.9 ± 5.7 μm, 397.5 ± 6.9 μm, 154.8 ± 106.5 μm, respectively (Movie S3†). Since the red fluorescent particles were very close to the carrier flow (added fluorescent sodium), we neglected the wall restriction and assumed *β*_2_ = 1. Therefore, *β*_2_ for the green fluorescent particle is calculated as 0.372. From microscopic observation, it appears that jakobid cells become smaller in dynamic solution when the velocity of the sheath flow is increased, and jakobid cells tended to be parallel to the channel wall (Fig. S1b†) because of unbalanced forces. Thus, the critical dimension for the jakobid cell in dynamic situation should be the length of its minor axis. The value of *β*_2_ for jakobid cells was found to be 0.369 at least. Despite the fact that this *β*_2_ is slightly lower than our deduction, the test results still match our prediction, especially considering that the size and shape of individual jakobid cells cannot be identical to those of particles, and the measuring error of critical size would be quadratically magnified. Therefore, the resulting equation could offer a good reference to predict the cells' position after separation (*i.e.*, the receiving outlet, Fig. 2) and help in designing outlet configuration.

**Fig. 6:**
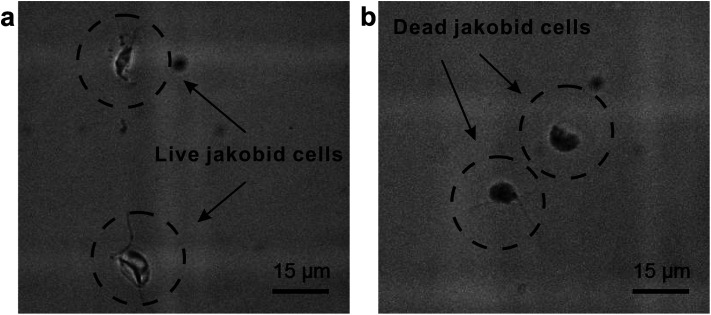
Activity detection: (a) collected jakobid cells mixed with the methylene blue; (b) dead jakobid cells killed by formaldehyde mixed with the methylene blue.

The second critical aspect we were concerned with in live cell separation is cell viability. In order to estimate cell survivability of collected jakobids, samples from the outputs were stained with methylene blue (Fig. 6a) and compared with dead jakobid cells killed with formaldehyde (Fig. 6b). Under magnification, the collected jakobid cells looked thinner and more transparent than the dead cell, which were rounder and much darker blue in color. A 5 μl aliquot of collected cells deposited onto a glass slide, showed few cells taking up the blue stain (Fig. S3†) and cell counting showed that cell survivability was above 90%. Collected cells, mixed with few bacteria, grew slowly but still showed reproductive capacity in fresh medium, when compared with similar numbers of cultured cells under the same conditions (Table S1†).

### 4.3 Cell separation with flow cytometry

Flow cytometry has been widely used for sorting cells of various types, both prokaryotes and eukaryotes. In order to get pure samples and large numbers of jakobid cells (Fig. S4†), tests were done under different temperatures and pressures on a mixed culture of living jakobid cells and their bacterial food. At 4 °C, 66.2% of bacteria were removed, resulting jakobid cells with increased purity from 11.0% to 26.8% of total output (Fig. S4a and c†). However, long exposure times to cold temperature could cause jakobid cells to enter a dormant stage, which can be difficult to break in the lab. Therefore, it is more difficult to accumulate a large number of pure jakobid cells with high viability over a certain time period (*e.g.* > 30 min) using flow cytometry. We also tested antibiotic treatment for removing bacteria, by adding both lysozyme and lactoferrin to the jakobid culture 1 hour before the flow cytometry cell sorting. This was technically easy and did not involve complicated experimental procedure, but the resulting purity of jakobid cells actually decreased (Fig. S4d†), attributing to the remains of bacteria and loss of jakobid cells in the supernatant. Two possible reasons for the low recovery efficiency of jakobid cells from collected supernatant might be: (1) antibiotic may cause the activity of jakobid cells to decrease and without active swimming the cells tended to fall to the bottom along with the dead bacteria. (2) the jakobids may actively swim towards the bottom where there are larger numbers of their bacterial food, leaving a small number of jakobid cell in the supernatant. However, in our microfluidic chip, the cells' taxis for food could be neglected when the hydrodynamic force is so strong that cells cannot swim freely. Thus, the separation efficiency of flow cytometry should be much lower than that of the microfluidic chip (see above). Analysis of the supernatant from the flow cytometry showed that only a small number of jakobid cells were obtained, and they only made up 7.1% of total number of jakobid cells and bacteria.

Thus, the microfluidic separation method clearly outperformed the conventional biotic separation technique in this study. This conclusion is based on the following reasons. (1) The microfluidic method is less harmful to fragile cells due to its low pressure (<0.8 psi), soft interaction with fluids, and no extra stimulus. The latter is important as stimuli such as antibiotics, light, or unsuitable temperature, may negatively affect biological activity for sensitive cells. (2) A higher purity of jakobid cells was obtained from the microfluidic method than from the flow cytometry with over 94% of bacteria successfully removed. (3) Unlike conventional biotic methods that are used for general cell fractionation with only a low requirement of purity, microfluidic chips are tailor-designed according to the size and distribution patterns in liquid of target cells. Such a high adjustability should allow higher separation efficiency and recovery efficiency. (4) Using conventional separation methods generally demand more biological knowledge towards target cells, such as the complexity of cells' internal structures which enable gate setting in separation based on a flow cytometry or functions of various antibiotics which help the separation based on antibiotic treatment.

## 5. Conclusion

Using a steep velocity gradient induced soft inertial force, microfluidic separation of jakobid cells from *Klebsiella* sp. showed remarkably higher purity and viability than the conventional biotic separation technique. Due to the simple operation, capability to separate asymmetric cells, and robust separation performance, this microfluidic separation method could be used to purify a variety of target cells from complex environments.

## Conflicts of interest

There are no conflicts to declare.

## Supplementary Material

RA-008-C8RA05328F-s001

RA-008-C8RA05328F-s002

RA-008-C8RA05328F-s003

RA-008-C8RA05328F-s004
