# Supplementary material for: High purity and viability cell separation of a bacterivorous jakobid flagellate based on a steep velocity gradient induced soft inertial force
Source: RSC Adv. 2018 Oct 16;8(62):35512–20. doi: 10.1039/c8ra05328f (PMC9087867; doi:10.1039/c8ra05328f)
Supplement: RA-008-C8RA05328F-s004 [file RA-008-C8RA05328F-s004.pdf]

Electronic Supplementary Material (ESI) for RSC Advances.
This journal is © The Royal Society of Chemistry 2018

Electronic Supplementary Information

High purity and viability cell separation of a bacterivorous jakobid flagellate based on a steep velocity gradient induced soft inertial force

Pan Deng,[a] Cheng-Jie Fu[*b] and Zhigang Wu[*ac]

[a]State Key Laboratory of Digital Manufacturing Equipment and Technology, Huazhong University of Science and Technology, Wuhan, China. Email: zgwu@hust.edu.cn; zhigang.wu@angstrom.uu.se

[b]Department of Organismal Biology, Uppsala University, Uppsala, Sweden

[c]Department of Engineering Sciences, The Ångström Laboratory, Uppsala University, Uppsala, Sweden

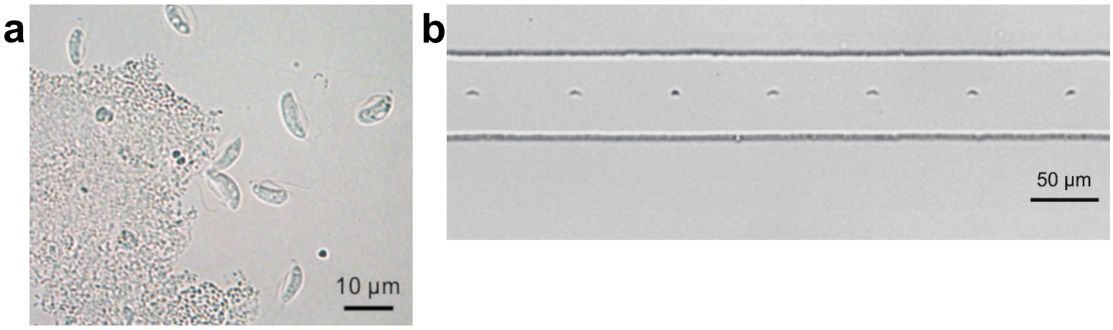

Fig. S1. Morphological images of jakobid cells. (a) Monoxenic culture of the jakobid S. ecuadoriensis cells under static environment. (b) 7 movement states of a single jakobid cell under different time in the main channel at sample flow rate 5 μl/min, two sheath flow (PBS) rate 15 μl/min.

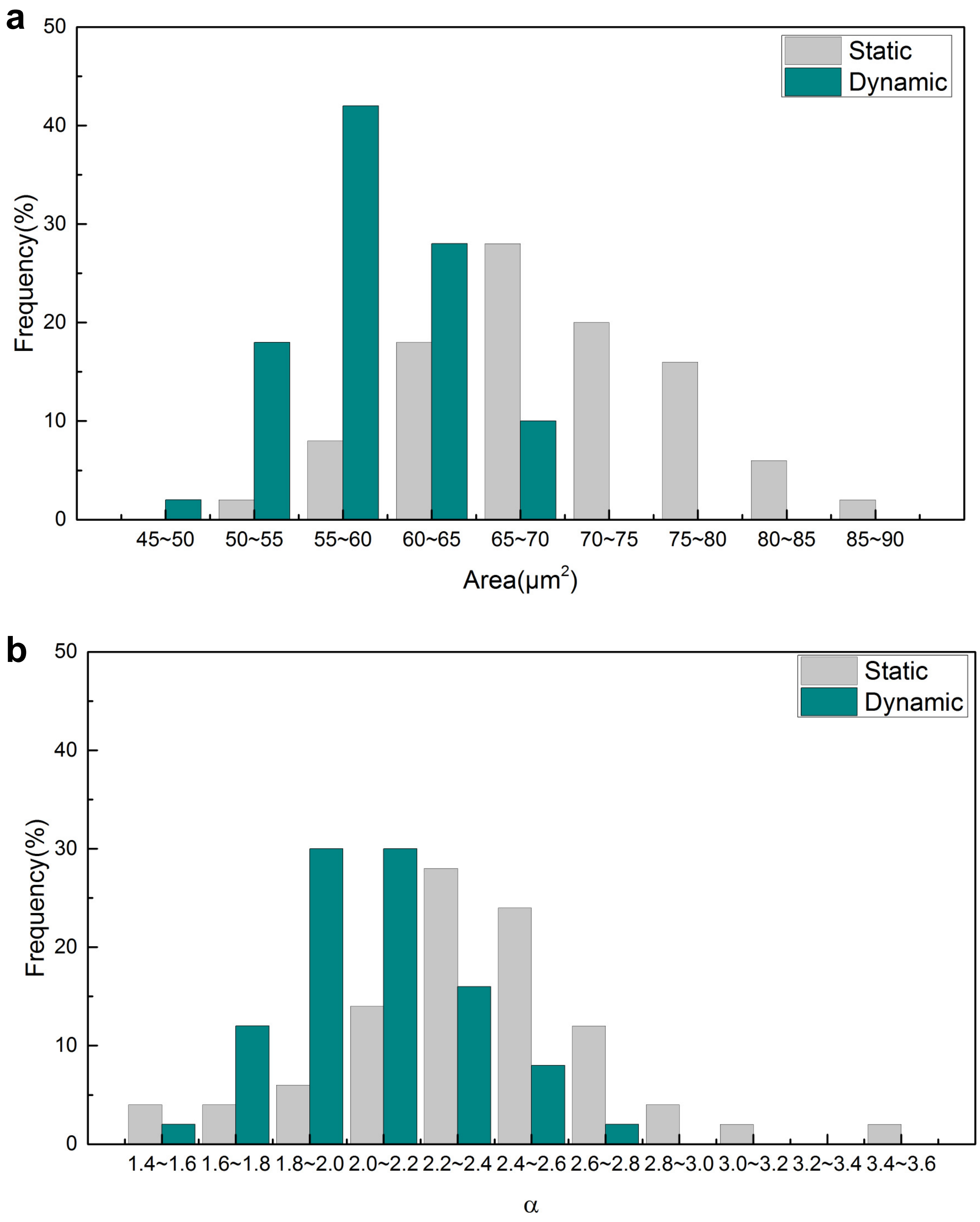

Fig. S2. Dimensional changes of jakobids' size (a) Planar mapping area distribution of jakobid cells under static environment and dynamic flow environment (sample flow rate 5 μl/min, two sheath flow (PBS) rate 15 μl/min). (b) Ratio of long axis to minor axis distribution of jakobid cells under static environment and dynamic flow environment (N=20).

Table S1. Density of recultured jakobid cells

	Density of collected cells (10^5/ml)	Density of cultured cells (10^5/ml)
3rd Day	0.4±0.1	2.5±0.1
4th Day	2.5±0.5	3.0±0.1
5th Day	4.0±0.5	4.1±0.4
6th Day	4.3±0.8	4.5±0.7
7th Day	5.2±0.7	4.5±0.5
8th Day	5.0±0.6	4.4±0.4

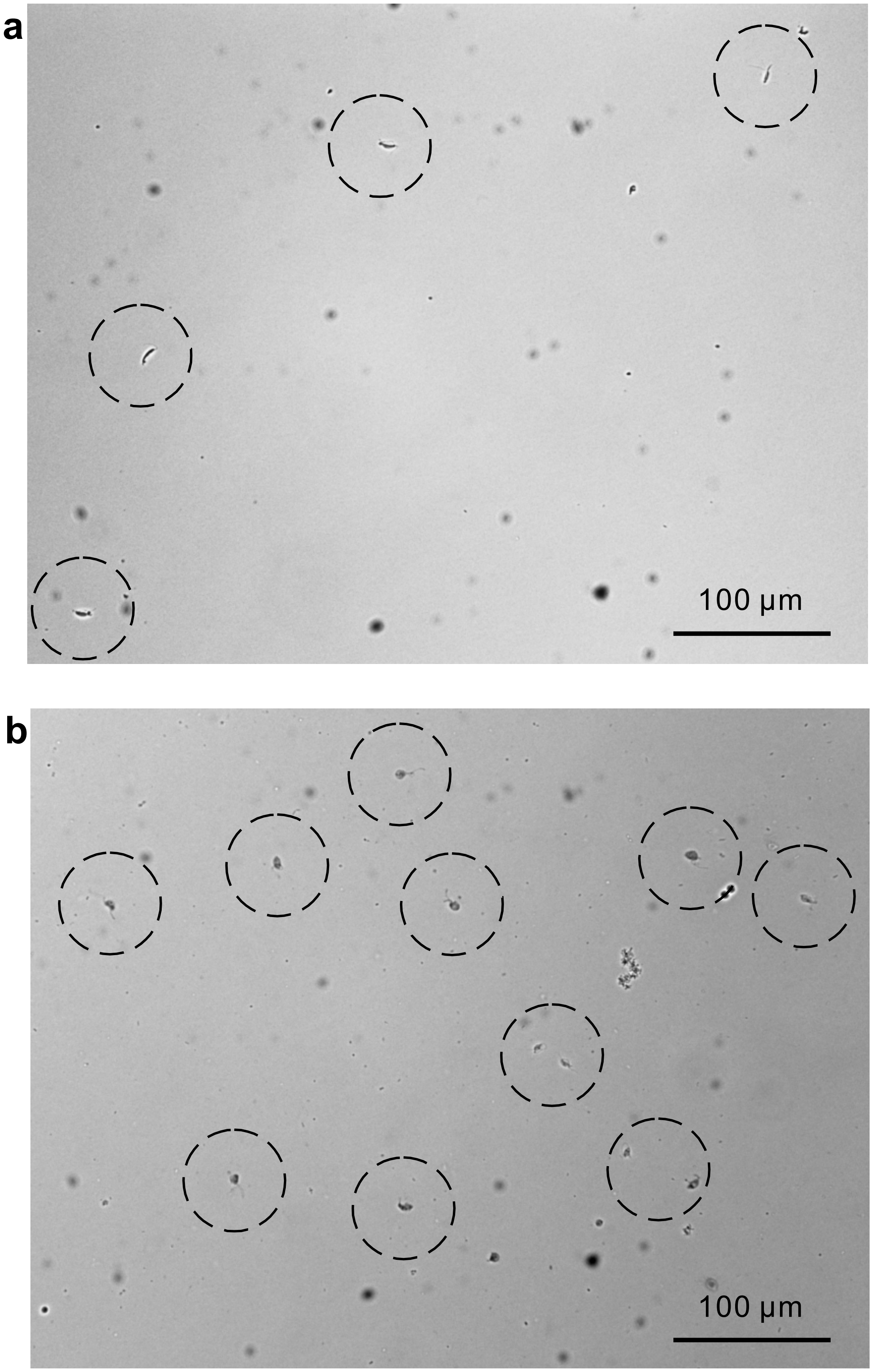

Fig. S3. Images of 5 μl droplet on a glass slide, which containing methylene blue and collected jakobid cells (a) or dead jakobid cells killed by formaldehyde (b).

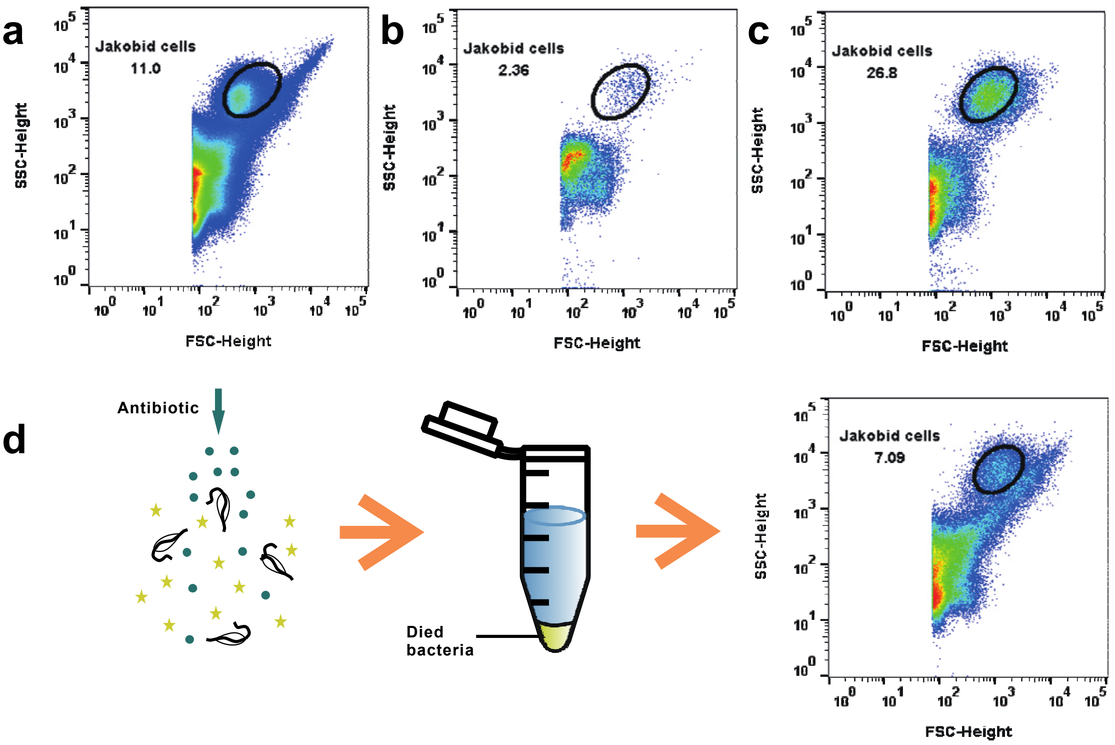

Fig. S4. Results of separation using conventional techniques (calculated in percentage terms): The jakobid cells and bacteria were analyzed and quantified at 25 psi by a flow cytometry showing the mixture before the sorting (a), after the flow cytometer sorting at room temperature (b), and at 4 °C (c). Antimicrobial treatment with bacteria for 1 hour and the supernatant was measured by a flow cytometry (d)

Jakobid cells cannot bear the normal pressure of the flow cytometry (60 psi) and lots of cell debris was found under the microscope, so the pressure was reduced and better results were got at 25 psi. Considering that room temperature is the optimum growth temperature for jakobid cells, the separation environment was set at room temperature, but the density of collected jakobid cells was much lower than that of sample solution (Fig. S5a, b).

Supplementary movie captions:

Movie S1. The movie shows that large particles separated from small particles in the broadened segment. It was played in a slow motion (play speed: 125× slower).

Movie S2. The movie shows the process of collecting separated particles in the broadened segment near the outlets. It was played in a slow motion (play speed: 100× slower).

Movie S3. the movie shows the displacements of particles and jakobid cells in the broadened segment. It was played in a slow motion (play speed: 500× slower).
